# Editorial: Proteostasis in central nervous system disorders

**DOI:** 10.3389/fnmol.2024.1394171

**Published:** 2024-03-18

**Authors:** An Zhou, Fang Bian

**Affiliations:** ^1^Department of Neurobiology, Neuroscience Institute, Morehouse School of Medicine, Atlanta, GA, United States; ^2^Department of Bioengineering, Office of Research and Innovation, The University of Texas at Dallas, Richardson, TX, United States

**Keywords:** neurological disorder, protein synthesis, proteomics, brain cell type-specific, synaptic, alpha-synuclein, biomarkers, personalized medicine

Maintaining proteostasis is essential for the CNS to function properly and to afford appropriate stress responses, as it is for other organs. The nerve tissues' high demand for energy metabolism and the unique architecture of neurons, notably extensive processes and synapses that can be distant from the soma, make CNS proteomes particularly sensitive to perturbation in protein biosynthesis or degradation activities. CNS proteomes comprise proteins from various types of cells, each with distinct proteomic compositions and stress-response mechanisms. Compounding the complexity, CNS proteomes are highly dynamic due not only to protein synthesis, modification, and degradation activities but also to the secretion of proteins and peptides from both neuronal and non-neuronal cells, which may occur constitutively or in regulated manners.

The Research Topic titled “*Proteostasis in CNS disorders*” in the Journal of Frontier of Molecular Neuroscience, within the “Brain Disease Mechanisms” section, presents recent discoveries that highlight, mechanistically, the dependence of neurophysiology of cortical neurons on protein synthesis (Cohen et al.), and how the proteomes of brain cells are altered in a cell type-specific manner in disease conditions (Sangster et al.). The topic offers an opinion article on an original study that entails a potentially novel mechanism underlying the transmission or spreading of disease-associated proteins (Taha et al.). A new technique for visualizing inter-neuron transportation of a cytoskeleton-associated protein in the brain is also introduced (Avallone et al.). Lastly, the topic includes a comprehensive review of emerging trends in neuroproteomics, with a focus on traumatic brain injury (TBI) research (Kobeissy et al.). The review discusses the potential benefits of incorporating artificial intelligence in the development of personalized medicine.

It is well-established that abnormalities in a single protein can lead to profound neurological disorders. For instance, Huntington's Disease is characterized by aggregated mutant huntingtin proteins. In the case of Fragile-X Syndrome, the lack of Fragile X Messenger Ribonucleoprotein (FMRP) due to the inherited silencing of the Fragile X messenger ribonucleoprotein 1 gene results in synaptic dysregulation and is ultimately manifested as learning and cognitive impairments; FMRP is an RNA-binding protein crucial for transporting a set of mRNAs needed for local protein synthesis at dendritic sites. The impact of pathophysiological conditions involving global attenuation in protein synthesis remains poorly understood, leaving many questions unanswered. For instance, in the brain, which synaptic properties are most susceptible to proteome-wide disruption in protein synthesis? Without a continuous supply of newly synthesized proteins, are there specific proteins that will be lost selectively and more quickly than others? Answers to these questions may shed light on what could constitute the so-called “failure point” in the neuronal response to disruptions, even transient ones, in protein synthesis.

Cohen et al. in their article “Synapse integrity and function: Dependence on protein synthesis and identification of potential failure points,” introduced a series of experiments in which the effects of inhibition of protein synthesis on neurophysiological properties were examined. They also conducted proteomic analysis of metabolically labeled cortical proteomes to identify proteins lost early upon protein synthesis inhibition. They reported that, in primary cultures of rodent cortical neurons, “*acute suppressions of protein synthesis are followed within hours by reductions in spontaneous network activity levels, impaired oxidative phosphorylation and mitochondrial function, and, importantly, destabilization and loss of both excitatory and inhibitory postsynaptic specializations*.” Further, proteomic analysis revealed that “*early-lost*” proteins included those related to synapse stability regulation, lipid metabolism, and bioenergetics. Interestingly and unexpectedly, they found proteins involved in Alzheimer's disease pathology also among early-lost proteins. These findings highlight, as noted by the authors, “*neuronal excitability, energy supply and synaptic stability as early occurring failure points under conditions of compromised supply of newly synthesized protein copies*.” It is worth mentioning that, in their proteomic analysis of metabolically labeled proteins, in reference to results from previously published studies (Cohen and Ziv, 2019; Cohen et al., 2020), the authors were able to distinguish early-lost proteins identified in this study from those that are known to have a shorter half-life. Though using exogenous protein synthesis inhibitors does not completely reconstitute compromised protein synthesis seen under pathophysiological conditions *in vivo*, the findings reported in this work may assist in understanding pathologies of several neurological disorders that incur acute, global disruption in protein synthesis, such as ischemic stroke, TBI and epilepsy.

There has been increased awareness and interest in understanding inter-neuron protein transportation through non-canonical routes like extracellular vesicles (EVs). While changes in local proteostasis are expected to occur, the roles of EVs in CNS disease pathologies are far from being fully understood. In an opinion article titled “A minute fraction of a-synuclein in extracellular vesicles may be a major contributor to alpha-synuclein spreading following autophagy inhibition” (Taha et al.), Taha et al. discussed a recent publication by Oh and colleagues entitled “S-Nitrosylation of p62 inhibits autophagic flux to promote alpha-synuclein secretion and spread in Parkinson's disease and Lewy body Dementia” (Oh et al., 2022). The multi-functional protein p62, an autophagy receptor protein and a known component of Lewy bodies, plays critical roles in autophagy. In Oh et al. (2022) paper, the authors conducted a series of sophisticated molecular and cell biological experiments using human iPSC-derived neurons, providing evidence that “*nitrosylation of p62 leads to inhibition of autophagy, which in turn increases the extracellular release of alpha-synuclein*.” This sheds light on a new aspect of the mechanism underlying synucleinopathies. In discussing the significance of the work by Oh et al. (2022), Taha et al. proposed intriguing questions for future studies, such as the potential involvement of SNO-p62 in proteinopathies characteristic of neurodegenerative diseases.

Avallone et al. in the article “Visualizing Arc protein dynamics and localization in the mammalian brain using AAV-mediated *in situ* gene labeling,” introduced a novel technique that allows for the visualization of protein dynamics and localization in the brain *in vivo*. Using this method, the authors demonstrated the transfer of cytoskeleton-associated protein Arc between post- and pre-synaptic sites, providing the first *in vivo* evidence for Arc's inter-neuron transfer in the mammalian brain. From the perspective of proteostasis maintenance and regulation, this study prompts consideration of how the transfer of post-synaptic proteins like Arc may alter local, pre-synaptic proteomes and vice versa.

The heterogeneity in brain cell types and subtypes limits the utility of omics data from tissue-level analyses. There has been increasing demand for identifying cell type-specific changes in the pathologies of CNS diseases. Mucolipidosis IV (MLIV) is a rare, severe disorder caused by mutations in the MCOLN1 gene. Phenotypically, this disorder is characteristic of lysosomal dysregulation primarily affecting CNS, with a brain pathology consistent with hypomyelinating leukodystrophies. From a pathogenic point of view, much remains unknown for MLIV. In their article titled “Brain cell type specific proteomics approach to discover pathological mechanisms in the childhood CNS disorder mucolipidosis type IV” (Sangster et al.), Sangster et al. reported results from proteomic analysis of several brain cell populations freshly sorted from brains of Mcoln1–/– mice, including neurons, astrocytes, oligodendrocytes, and neural stem cells. They noted that the data reconstituted molecular signatures of MLIV and identified dysregulated pathways specific to the analyzed cell populations, providing hope that these results will guide the identification of cell-specific biomarkers and therapeutic targets for MLIV.

In the latest article of this Research Topic, Kobeissy et al. provided a comprehensive review on topic of “Advances in neuroproteomics for neurotrauma: unraveling insights for personalized medicine and future prospects.” Taking advantage of their extensive experience in neurotrauma (TBI) research using various proteomic approaches, the authors reviewed and discussed the advantages and limitations of several major neuroproteomics techniques; these techniques include shotgun proteomics with or without labeling, glycoproteomics, peptidomics, single-cell proteomics, chip-based techniques, spatial-temporal protein distribution by mass spectrometry imaging, and high-throughput immunoblotting adopted in neurodegenerative disease research. Precision medical treatments for TBI are currently limited, and neuroproteomics studies in clinical trials primarily rely on sampling various biofluids. Appreciably, this review article includes several graphic illustrations describing the principal approaches or workflows for neuroproteomics studies. The need for personalized neurotrauma treatment necessitates a multivariate factorial approach using machine learning and artificial intelligence to predict, correlate, and map multivariate factors in TBI. At length, by reviewing published studies involving either human patients or animal models, the authors summarized and compared studies using various machine learning tools. In summation of expert opinions, Kobeissy et al. highlight the essentialness of bioinformatic and statistical approaches in assisting meaningful interpretation and disentangling of large volume high-throughput data, and challenges in defining and standardizing methodologies and protocols involved in neuroproteomics research, from sampling of biospecimen through data analysis.

## Author contributions

AZ: Conceptualization, Writing—original draft, Writing—review & editing. FB: Writing—original draft, Writing—review & editing.
